# *CORL* Expression and Function in Insulin Producing Neurons Reversibly Influences Adult Longevity in *Drosophila*

**DOI:** 10.1534/g3.118.200572

**Published:** 2018-07-13

**Authors:** Nancy L. Tran, Samuel L. Goldsmith, Agapi Dimitriadou, Norma T. Takaesu, Christos Consoulas, Stuart J. Newfeld

**Affiliations:** * School of Life Sciences, Arizona State University, Tempe AZ 85287-4501; † Medical School, National and Kapodistrian University of Athens, Athens, Greece

**Keywords:** Fussel/SKOR, dILP2, Drifter, pars intercerebralis, lifespan extension

## Abstract

CORL proteins (known as SKOR in mice, Fussel in humans and fussel in Flybase) are a family of CNS specific proteins related to Sno/Ski oncogenes. Their developmental and adult roles are largely unknown. A Drosophila *CORL* (dCORL) reporter gene is expressed in all Drosophila insulin-like peptide 2 (dILP2) neurons of the pars intercerebralis (PI) of the larval and adult brain. The transcription factor Drifter is also expressed in the PI in a subset of dCORL and dILP2 expressing neurons and in several non-dILP2 neurons. *dCORL* mutant virgin adult brains are missing all dILP2 neurons that do not also express Drifter. This phenotype is also seen when expressing dCORL-RNAi in neurosecretory cells of the PI. *dCORL* mutant virgin adults of both sexes have a significantly shorter lifespan than their parental strain. This longevity defect is completely reversed by mating (lifespan increases over 50% for males and females). Analyses of *dCORL* mutant mated adult brains revealed a complete rescue of dILP2 neurons without Drifter. Taken together, the data suggest that *dCORL* participates in a neural network connecting the insulin signaling pathway, longevity and mating. The conserved sequence and CNS specificity of all CORL proteins imply that this network may be operating in mammals.

The majority of secreted Transforming Growth Factor-β (TGF-β) family members belong to either the Decapentaplegic (Dpp)/Bone Morphogenetic Protein (BMP) or the TGF-β/Activin subfamilies. Both subfamilies perform a myriad of tasks during development and homeostasis while mutations disrupting TGF-β pathways can lead to disease. One mechanism of regulating TGF-β functions is modulation of Smad signal transducer activity. An important group of Smad interacting proteins are the Sno/Ski family of oncogenes. Sno/Ski proteins play positive or negative roles in TGF-β signaling depending upon the cellular context. They can also serve as a pathway switch that simultaneously facilitates TGF-β/Activin signaling while antagonizing Dpp/BMP signaling (Takaesu *et al.* 2006). In addition, Drosophila *Snoo* (also known as *dSno*) has a role outside of TGF-β signaling as an antagonist of Wg signaling (Quijano *et al.* 2010).

mCORL1 (mSKOR1) was identified as a Sno/Ski family member that functions as a transcriptional co-repressor. In embryos mCORL1 is expressed only in dorsal interneurons of the cerebellum (Mizuhara *et al.* 2005). Developmental studies of mCORL2 (mSKOR2) showed that it is expressed only in Purkinje neurons of the cerebellum (Minaki *et al.* 2008). Loss of function studies of mSKOR2 revealed a requirement for Purkinje cell differentiation (Miyata *et al.* 2010; Wang *et al.* 2011). mSKOR2 knockouts demonstrated that this is accomplished by inhibiting interneuron fate (Nakatani *et al.* 2014). No knockout studies of mSKOR1 have been reported. mSKOR1 is primarily, though not exclusively, expressed in the cerebellum of adults while mSKOR2 expression is restricted to the cerebellum in adults (Yue *et al.* 2014).

There are two human Fussel proteins. Fussel15 is homologous to mSKOR1 and Fussel18 is homologous to mSKOR2. The Fussel15 expression pattern is conserved with mSKOR1. It is present primarily in the adult cerebellum. There are also low levels of transcription in the lung and small intestine. The Fussel18 expression pattern is conserved with mSKOR2. It is restricted to the adult cerebellum (Fagerberg *et al.* 2014). Genome-wide association studies have linked mutations in Fussel15/hSKOR1 to two ataxias (*e.g.*, Li *et al.* 2017). These ataxias are thought to result from dysfunction in the cerebellum, as that is the site of movement in the brain. No syndromes are yet associated with mutations in Fussel18/hSKOR2.

In the only study of a *dCORL* mutation, one aspect of its larval brain expression was shown to function downstream of the TGF-β/Activin receptor Baboon in the transcriptional activation of Ecdysone Receptor-B1 in the mushroom body. In parallel, biochemical analyses of mCORL1 revealed a physical interaction with mSmad3 but not other Smads. Taken together the genetic and biochemical data suggested that dCORL is a Smad-interacting protein that facilitates TGF-β/Activin subfamily signaling in the larval mushroom body (Takaesu *et al.* 2012). Other functions of *dCORL* remain unknown.

Three other genes with expression in the larval and adult brain are Drifter, dILP2 and dILP5. Drifter is a transcription factor (also known as ventral veinless) that contains both POU and Homeobox domains. It plays numerous roles during development of the CNS including in medullary neurons of the larval optic lobe (Hasegawa *et al.* 2011) and projection neurons of the adult antennal lobe (Komiyama and Luo 2007). Interestingly, Drifter’s closest human relative is Oct9/Brn4. This is a CNS specific transcription factor that plays a role in neuronal differentiation of the cochlea. Mutations in Oct9/Brn4 cause an X-linked form of hearing loss (de Kok *et al.* 1995). Drosophila insulin-like peptides 2 and 5 (dILP2 and dILP5) are secreted hormones with roles in metabolism, growth and longevity (Rulifson *et al.* 2002; Droujinine and Perrimon 2016). In the larval and adult brain both proteins are detected exclusively in neurosecretory cells of the PI (Broughton *et al.* 2005). In these cells dILP5 is directly activated by the transcription factor Dachshund (Dac; Okamoto *et al.* 2012).

Here we identify insulin producing neurons of the larval and adult PI as sites of *dCORL* expression and function. In *dCORL* mutant larval and virgin adult brains we found that all dILP2 neurons that do not also express Drifter are absent. This phenotype is also seen when expressing dCORL-RNAi in the PI. *dCORL* mutant virgin adults of both sexes have a significantly shorter then normal lifespan, but this defect is completely reversed by mating (lifespan increases over 50% for males and females). *dCORL* mutant mated adult brains analyzed at three and fifteen days old contain a completely rescued complement of dILP2 neurons lacking Drifter. Overall the data suggest the existence of a neural network connecting *dCORL*, the insulin signaling pathway, longevity and mating.

## Material and Methods

### Drosophila genetics

Fly stocks are as described: AH.lacZ (Tran *et al.* 2018), *Df(4)dCORL* (Takaesu *et al.* 2012), OK107.Gal4 (Enell *et al.* 2010), *Glu-RA^112b^* and *Glu-RA^2b^* (Bogdanik *et al.* 2004), *Pbac{RB}^e02096^*, *Pbac{WH}^f07015^* and *Pbac{WH}^f06253^* (Exelixis collection at Harvard Medical School; Takaesu *et al.* 2012), *sphinx^720RW^* (Dai *et al.* 2008), UAS.dCORL-RNAi (Takaesu *et al.* 2012) and *yw* (*y^1^w^67c23^* employed as wild type throughout). *Glu-RA^112^* and *sphinx^720R^* are demonstrated null alleles. *Pbac{RB}^e02096^* is expected to disrupt all predicted transcripts of CG32016 and thus would also serve as a null. For antibody staining larvae that have stopped wandering but not yet begun pupariation (prior to anterior spiracle eversion - equivalent to 122 hr at 25° for *yw*) were picked individually, sorted by sex and their CNS dissected in groups of 9-12. Adult virgins were collected either prior to sexual maturation, segregated by sex and aged to one, five and ten days post-eclosion, or aged three and fifteen days post-eclosion with males and females together. Brains were dissected in groups of 9-12. Counting neurons in the PI was conducted by scrolling through slices in ImageJ. For longevity assays, two protocols were used. Data reported in Tables 1 and 2 are derived from 100 flies per trial, housed as ten flies per vial (male or female virgins or males+females). Data reported in Tables S1 and S2 are derived from 16 flies per trial, housed as four flies per vial (male or female virgins) or four sibmated pairs per vial. In all cases, vials were observed daily and each death was noted until none survived. Any animal that had a nonage-related death (*e.g.*, stuck in food or lost) was censored. Flies were transferred into new vials with fresh food every other day.

**Table 1:** *dCORL* mutant virgin adult longevity defects are fully rescued by mating in both sexes.^a^ T-test statistics^b^

Genotype	Mean Lifespan	*P* value	Genotype	Mean Lifespan	Δ	*P* value	
*Df(4)* virgin female	19.5+/−3.4		*Df(4)* mated female	33.0+/−6.7	**+ 64**%	<1.0x10^−6^	Mated longer
*yw*^c^ virgin female	31.7+/−7.0		*yw* mated female	33.9+/−7.3	+ 07%	0.14	No diff
*Df(4)* virgin female	19.5+/−3.4	$\frac{\text{vs}.\;Df\mathrm{(4)}}{<1.0{\text{x1}0}^{-6}}$	*Df(4)* mated female	33.0+/−6.7		$\frac{vs.\;Df(4)}{0.57}$	
*yw* virgin female	31.7+/−7.0		*yw* mated female	33.9+/−7.3			
for virgins: *yw* significantly longer than *Df(4)*				for mated: no difference *yw* and *Df(4)*			
*Df(4)* virgin male	23.2+/−3.5		*Df(4)* mated male	35.0+/−7.1	**+ 51**%	<1.0x10^−6^	Mated longer
*yw* virgin male	38.3+/−6.8		*yw* mated male	36.4+/−7.7	−05%	0.08	No diff
*Df(4)* virgin male	23.2+/−3.5	$\frac{\text{vs}.\;Df\mathrm{(4)}}{<1.0{\text{x10}}^{-6}}$	*Df(4)* mated male	35.0+/−7.1		$\frac{\text{vs}.\;Df(4)}{0.07}$	
*yw* virgin male	38.3+/−6.8		*yw* mated male	36.4+/−7.7			
for virgins: *yw* significantly longer than *Df(4)*				for mated: no difference *yw* and *Df(4)*			

a. Data set of eight experiments including six additional controls shown in Table S1.

b. All data were normally distributed per D’Agostino-Pearson omnibus test.

c. Note that *yw* is the genetic background of *Df(4)dCORL*.

**Table 2:** *dCORL* mutant virgin adult longevity defects are fully rescued by mating in both sexes. Χ^2^ and log-rank statistics

Χ^2^ value; log-rank test *P* value	*Df(4)* male mated	*Df(4)* female mated	*yw* male mated	*yw* female mated	Χ^2^ value; log-rank test *P* value	*yw* male virgin	*yw* female virgin	*yw* male mated	*yw* female mated
*Df(4)* male virgin	135.9 <1.0x10^−6^				*Df(4)* male virgin	169.0 <1.0x10^−6^			
*Df(4)* female virgin		158.3 <1.0x10^−6^			*Df(4)* female virgin		141.0 <1.0x10^−6^		
*yw* male virgin			1.6 0.21		*Df(4)* male mated			3.3 0.07	
*yw* female virgin				3.1 0.08	*Df(4)* female mated				0.75 0.39

### Statistics

Medians, means, standard deviations and pairwise p values employing Student’s T-test (independent, two tailed) were generated in Excel. D’Agostino-Pearson omnibus normality tests and Mantel-Cox log-rank tests were conducted with GraphPad Prism 6.00.

### Immunohistochemistry

AH.lacZ is a nuclear-lacZ reporter (high levels of expression yield nuclear and cytoplasmic staining) that contains a region of genomic DNA 7-11kb upstream of the *dCORL* transcription start as described (Tran *et al.* 2018). Two independent insertions were analyzed to eliminate position effects (lines 1A and 3A). For 3-color confocal detection of antibodies, tissues were fixed in 4% formaldehyde, rinsed and stored in methanol until staining. Primary antibodies were: rabbit and rat α-lacZ (Organon Teknika, Durham; MBL, Nagoya), guinea pig α-Toy (gift of Uwe Walldorf, Saarland Univ.), mouse α-Dac (DSHB 1-1-c), mouse α-Fas2 (DSHB 1D4), rat α-Elav (DSHB 7E8A10), mouse-α Repo (DSHB 8D12), guinea pig α-Drifter (gift of Makoto Sato, Kanazawa Univ.) and rat α-dILP2 (gift of Pierre Leopold, Nice Univ.). Secondary antibodies were: goat α-mouse, α-rabbit, α-guinea pig or α-rat Alexa Fluor 488, 546, and 633 (Molecular Probes). Tissues were imaged on Leica SP5 or SP8 confocal microscopes with slices acquired every 2µm.

### Data Availability

Strains are available upon request. The authors affirm that all data necessary for confirming the conclusions of the article are present within the article, figures, and tables. Supplemental material available at Figshare: https://doi.org/10.25387/g3.6837809.

## Results

### dCORL is expressed in all dILP2 neurons of the larval and adult brain

Previously we showed that *dCORL* displays CNS specific transcription in embryos and third instar larvae. Our clonal analysis of *dCORL* mutant larval brains identified a requirement for *dCORL* in TGF-β/Activin signal transduction in mushroom body neurons (Takaesu *et al.* 2012). Our recent analysis of reporter genes showed that *dCORL* is expressed in larval brain neurons but not larval mushroom body neurons (Tran *et al.* 2018). Taken together these results indicated that *dCORL* functions non-autonomously in the mushroom body. This led us to consider the possibility that *dCORL* is expressed in the nearby PI, a brain region where neurosecretory cells initiate the insulin signaling pathway.

**Figure 1:**
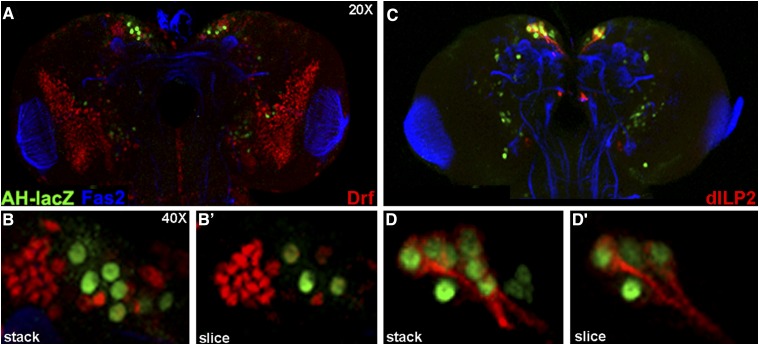
*dCORL* is expressed in all dILP2 neurons of the larval brain. Dorsal view of AH.lacZ transgenic larval female brains with anterior up. A) AH.lacZ (green-nuclear), Fas2 (blue–marks a distinct subset of neurons including the optic lobe) and Drifter (red-nuclear) at 20X. AH.lacZ is a nuclear-lacZ reporter containing genomic DNA upstream of *dCORL*. Expression of both lacZ and Drifter in the PI is seen. B) 40X stack and single slice of the PI in the left hemisphere from the same brain. Co-expression of AH.lacZ and Drifter in a subset of neurons is visible. C) AH.lacZ (green), Fas2 (blue) and dILP2 (red-cytoplasmic) at 20X. Coexpression of AH.lacZ and dILP2 in the PI is evident (neurons with red cytoplasm have green nuclei). Axons from the coexpressing neurons in both hemispheres extend medially. D) 40X stack and single slice of the PI in the left hemisphere from the same brain. Coexpression of AH.lacZ and dILP2 in all dILP2 neurons of the PI is confirmed (all neurons with red cytoplasm have green nuclei). An additional AH.lacZ neuron sits just medial to the co-expressing neurons. Note that the bottom four panels in Fig. 5 of Tran *et al.* (2018) are intentionally similar to the bottom four panels here. The point of the similarity is to document the logic connecting these papers.

Initial co-expression studies employing the *dCORL* reporter AH.lacZ and the transcription factor Drifter (Hasegawa *et al.* 2011) revealed overlap in a subset of neurons of the PI (roughly 4-5 per hemisphere; Figure 1A,B). Similar experiments with AH.lacZ and dILP2 (Rulifson *et al.* 2002) revealed that AH.lacZ is present in every dILP2 expressing neuron in the PI (range 6-8 per hemisphere; Figure 1C,D). There is a single AH.lacZ neuron located medially to the coexpressing neurons in each brain hemisphere. Coexpression with dILP2 bolstered our hypothesis of *dCORL* non-autonomous function in the larval mushroom body and suggested a role in the insulin signaling pathway.

**Figure 2:**
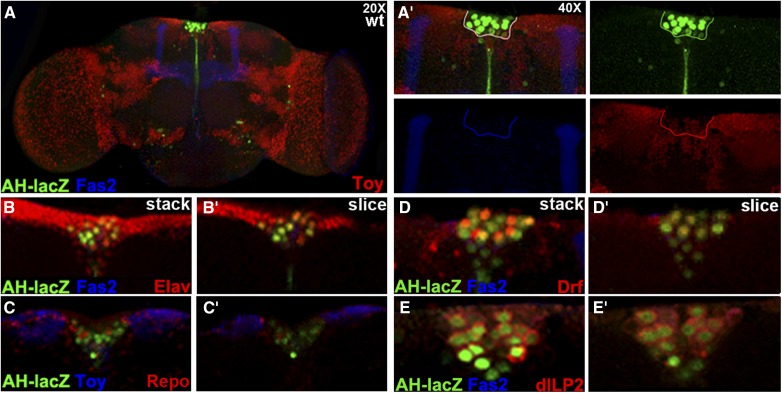
*dCORL* is expressed in all dILP2 neurons of the adult brain. One day old AH.lacZ transgenic virgin adult female brains in dorsal view with anterior up. A) AH.lacZ (green), Fas2 (blue marks mushroom body lobes employed to determine orientation) and Toy (red-nuclear) at 20X. AH.lacZ expression is clearly visible in the PI. A’) 40X views in 3-color and as single channels. An arbitrary line in the 3-color view surrounding the PI serves as a landmark in the individual channels. Strong AH.lacZ expression in neurons of the PI overflows the nucleus into the descending axon bundle. A few scattered neurons in the posterior display AH.lacZ expression, as do 2-3 neurons at the border of the optic lobe. No coexpression between AH.lacZ and Toy in the PI is noted. Toy expressing neurons are located ventral to AH.lacZ neurons and their nuclei are a different size. B) AH.lacZ (green), Fas2 (blue) and Elav (red-nuclear) show coexpression (yellow) in neurons of the PI. C) AH.lacZ (green), Toy (blue) and Repo (red-nuclear) shows no coexpression in glia of the PI (red and green neurons are adjacent). D) AH.lacZ (green), Fas2 (blue) and Drifter (red) shows coexpression of AH.lacZ and Drifter (yellow) in a subset of neurons within the PI. E) AH.lacZ (green), Fas2 (blue) and dILP2 (red) shows coexpression in all dILP2 neurons of the PI (all neurons with red cytoplasm have green nuclei). An additional AH.lacZ neuron without dILP2 is also visible in the PI of each hemisphere, sitting medially.

The presence of defects in the adult mushroom body of *dCORL* mutants (Takaesu *et al.* 2012), led us to examine *dCORL* reporter adult brain expression. We analyzed virgin adult females and males (one day old) utilizing AH.lacZ. We found that AH.lacZ is strongly expressed in the PI, a topologically recognizable region at the midline of the dorsal anterior-most section of the brain in both sexes (Figure 2A). Importantly, AH.lacZ in the adult PI does not reflect the expression of *twin of eyeless (toy)*, a divergently transcribed gene immediately distal to *dCORL* (Tran *et al.* 2018). Extending the study, no obvious differences were noted in AH.lacZ expression between female virgins at one, five and ten days old. Co-expression analyses of AH.lacZ with Elav and Repo in adults (Figure 2B,C) were consistent with those in larvae (Tran *et al.* 2018). AH.lacZ is found in neurons but not glia.

Coexpression studies of AH.lacZ and Drifter in the adult PI revealed that there are more neurons of both types in the adult than in larvae (AH.lacZ both hemispheres mean 18.4 neurons, n = 5; Drifter mean 9.8 neurons, n = 6; Figure 2D). Also in the adult, overlap is more extensive with 5-7 neurons co-expressing AH.lacZ and Drifter. Overall, there are 3-4 neurons expressing only AH.lacZ and the same number expressing only Drifter interspersed with the co-expressing neurons. An additional group of 4-6 AH.lacZ neurons form an inverted triangle pointing medially below the coexpressing neurons. We then examined dILP2 and AH.lacZ. We confirmed the report that dILP2 is expressed in a larger group of neurons in the adult PI then in larvae (Géminard *et al.* 2009). Consistent with our larval data, AH.lacZ is present in the nuclei of every dILP2 neuron (dILP2 mean 16.7 neurons, n = 6; Figure 2E). Again as in larvae, there is a single AH.lacZ neuron located medially to the coexpressing neurons in each brain hemisphere. Interestingly, the AH.lacZ pattern in the PI (every dILP2 cell plus one per hemisphere) is similar to a subset of the adult expression of Eyeless-Gal4 (OK107.Gal4; Enell *et al.* 2010).

### dCORL mutant larval & virgin adult brains are missing all dILP2 neurons lacking Drifter

**Figure 3:**
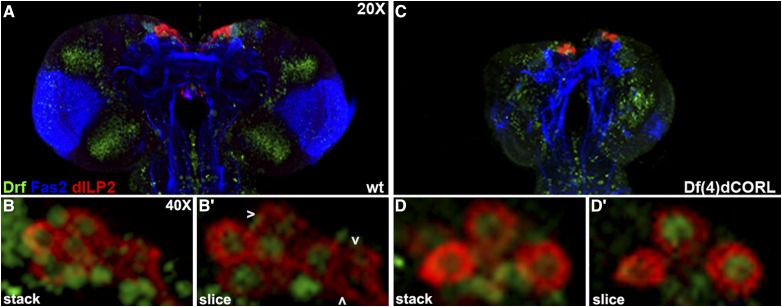
*dCORL* mutant larval brains are missing all dILP2 neurons lacking Drifter. Dorsal view of larval female brains with anterior up stained with Drifter (green - nuclear), Fas2 (blue) and dILP2 (red - cytoplasmic). A) Wild type (*y^1^w^67c23^*) at 20X shows the presence of dILP2 and Drifter in the PI. B) 40X stack and single slice of the PI of the left hemisphere from the same brain. The single slice view shows that the dILP2 neurons are essentially in a monolayer (all 8 neurons are visible in a single slice). There are neurons that express Drifter alone, three neurons expressing dILP2 alone (white arrowheads), and five neurons that express both. C) *Df(4)dCORL* brain (larvae was aged to the same developmental point as wild type before dissection) at 20X shown to scale. Notwithstanding the complete disarray of Fas2 and Drifter staining as well as the statistically significant reduction in *Df(4)dCORL* brain size, both dILP2 and Drifter are present in the PI. D) 40X stack and single slice of the PI of the left hemisphere from the same brain. The single slice view shows that there are just 5 dILP2 neurons (*vs.* 8 in wild type) and that their topology is altered. These are no longer in a monolayer (only 3 neurons are visible in a single slice). There are no neurons expressing dILP2 alone as all 5 dILP2 neurons express Drifter.

Given the coincidence in the PI of AH.lacZ, dILP2 and Drifter, we examined dILP2 and Drifter in the brains of larvae and virgin adults that were homozygous for a *dCORL* deletion (*Df(4)dCORL*; Takaesu *et al.* 2012). The goal was to determine if the loss of *dCORL* had any effect on dILP2 or Drifter expression. We began with third instar larvae. In wild type (*y^1^w^67c23^* throughout), we noted that dILP2 neurons form a monolayer (*i.e.*, all visible in the same slice) and that a subset of dILP2 neurons also expressed Drifter (five of eight in Figure 3A,B). There were dIPL2 only neurons (three) and several Drifter only neurons. In a *dCORL* mutant brain a statistically significant reduction in *Df(4)dCORL* brain size was noted (roughly 60% smaller than wild type) and the monolayer was disrupted (*i.e.*, not all dILP2 neurons are visible in the same slice). Surprisingly, all dILP2 neurons lacking Drifter were absent (Figure 3C,D). Neurons expressing Drifter, either with dILP2 or alone did not appear to be affected.

**Figure 4:**
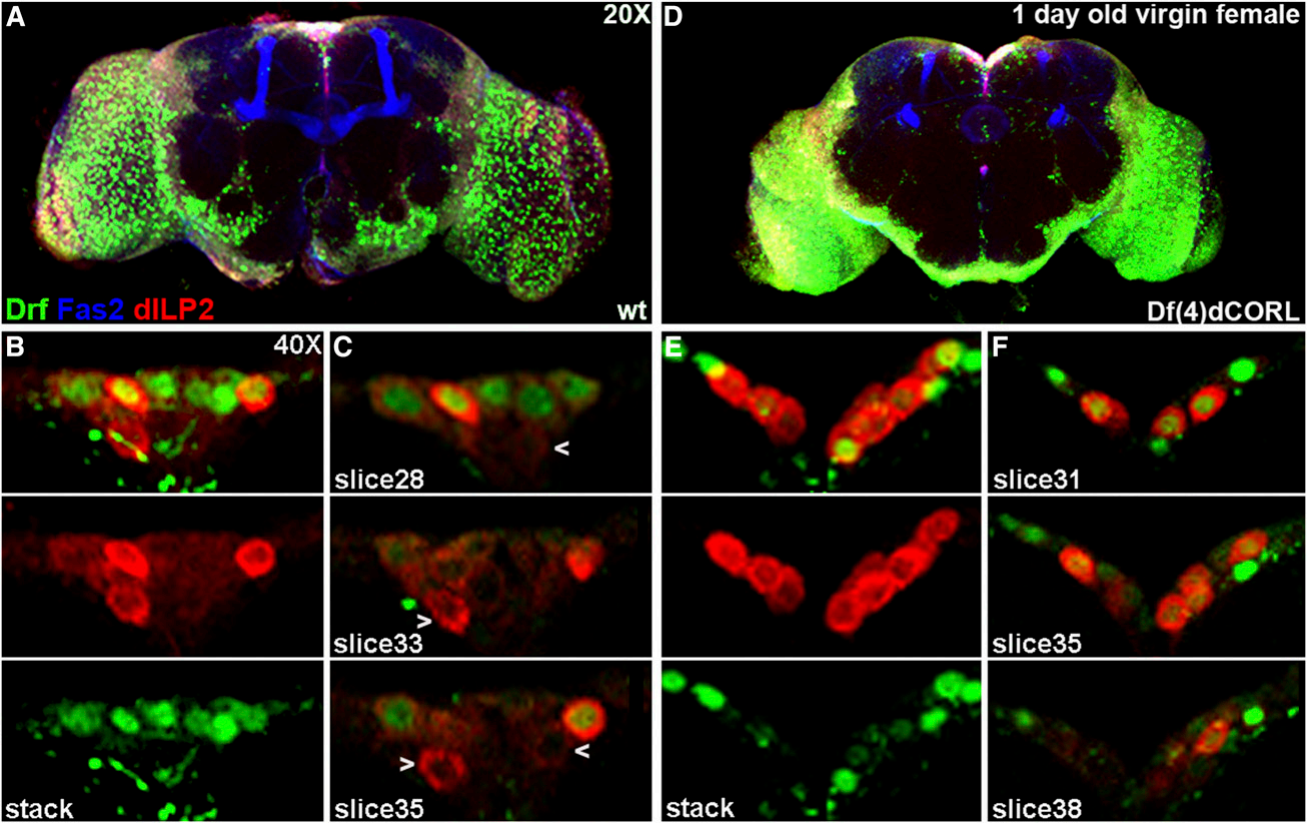
*dCORL* mutant virgin adult brains are missing all dILP2 neurons lacking Drifter. One day old virgin adult female brain in dorsal view with anterior up displaying Drifter (green), Fas2 (blue) and dILP2 (red). A) Wild type (*y^1^w^67c23^*) at 20X shows dILP2 and Drifter in the PI. B) 40X stack of the PI from the same brain: (top) 2-color, (middle and bottom) single channel of dILP2 (red) and Drifter (green). The 2-color view shows that dILP2-Drifter coexpressing neurons form a single straight row along the apical surface of the PI with additional dILP2 neurons forming an inverted triangle. There are neurons that express Drifter alone in the apical row. C) Three slices show the pyramidal structure of the PI (not all dILP2 neurons are visible in the same slice). Four dILP2 neurons that do not express Drifter are indicated (white arrowheads). D) *Df(4)dCORL* brain at 20X shown to scale with dILP2 and Drifter in the PI. E) 40X stack of the PI from the same brain: (top) 2-color, (middle and bottom) single channel of dILP2 (red) and Drifter (green). The 2-color view shows there has been a decrease in the number of dILP2 expressing neurons. The dILP2-Drifter coexpressing neurons still form a single row but the absence of additional dILP2 neurons medially caused the apical row to collapse into a V-shape. There are neurons that express Drifter alone in the dILP2-Drifter V-shape and their number has not changed. F) Three slices show that the V-shaped row of dILP2-Drifter neurons has a non-wild type structure. No dILP2 neurons that do not express Drifter are present.

We then compared the expression patterns of dILP2 and Drifter in one day old wild type virgin adult females. dILP2 and Drifter coexpressing neurons occupy a single layer along the apical surface of the PI with a set of dILP2 only neurons forming an inverted triangle pointing medially (Figure 4A,B). There are also occasional neurons that express Drifter alone in the dILP2-Drifter single layer. Analyzing eight individual slices covering a span of 16 microns confirmed the 3-dimensional structure of the adult PI. The dILP2-Drifter layer of neurons is the apical base of an inverted pyramid with the additional dILP2 only neurons pointed medially (Figure 4C). This is consistent with previous observations of AH.lacZ and Drifter in the same genotype - AH.lacZ only neurons form an inverted triangle pointing medially below a single layer of coexpressing neurons (refer back to Figure 2D).

Examining *dCORL* mutant brains from one day old virgin adult females, we found their size restored to essentially wild type (*e.g.*, roughly 85% for the example in Figure 4D). Further, both dILP2 and Drifter are present in the PI but there has been a 35% decrease in the average number of dILP2 expressing neurons (mean 11.4, n = 8; Figure 4D,E). This number is not significantly different from wild type due to the size of the standard error (Student’s T-test *P* = 0.07). In the *dCORL* mutant, the dILP2-Drifter coexpressing neurons still form a single layer along the apical surface of the PI but the absence of the pyramid of medially located dILP2 only neurons has caused this row to collapse into a V-shape rather than a straight line. There are still occasional neurons that express Drifter alone in the dILP2-Drifter row and the number of Drifter expressing neurons in the mutant is the same as wild type (mean 8.9, n = 8; *P* = 0.70). Analyzing individual slices covering 16 microns confirmed the absence of all medially located dILP2 only neurons in the adult PI of *dCORL* mutants (Figure 4F). The reduction in dILP2 neurons by 35% in *dCORL* mutant adults may seem modest but the fact that the missing neurons are exclusively dILP2 neurons lacking Drifter in both larvae and adults is striking.

**Figure 5:**
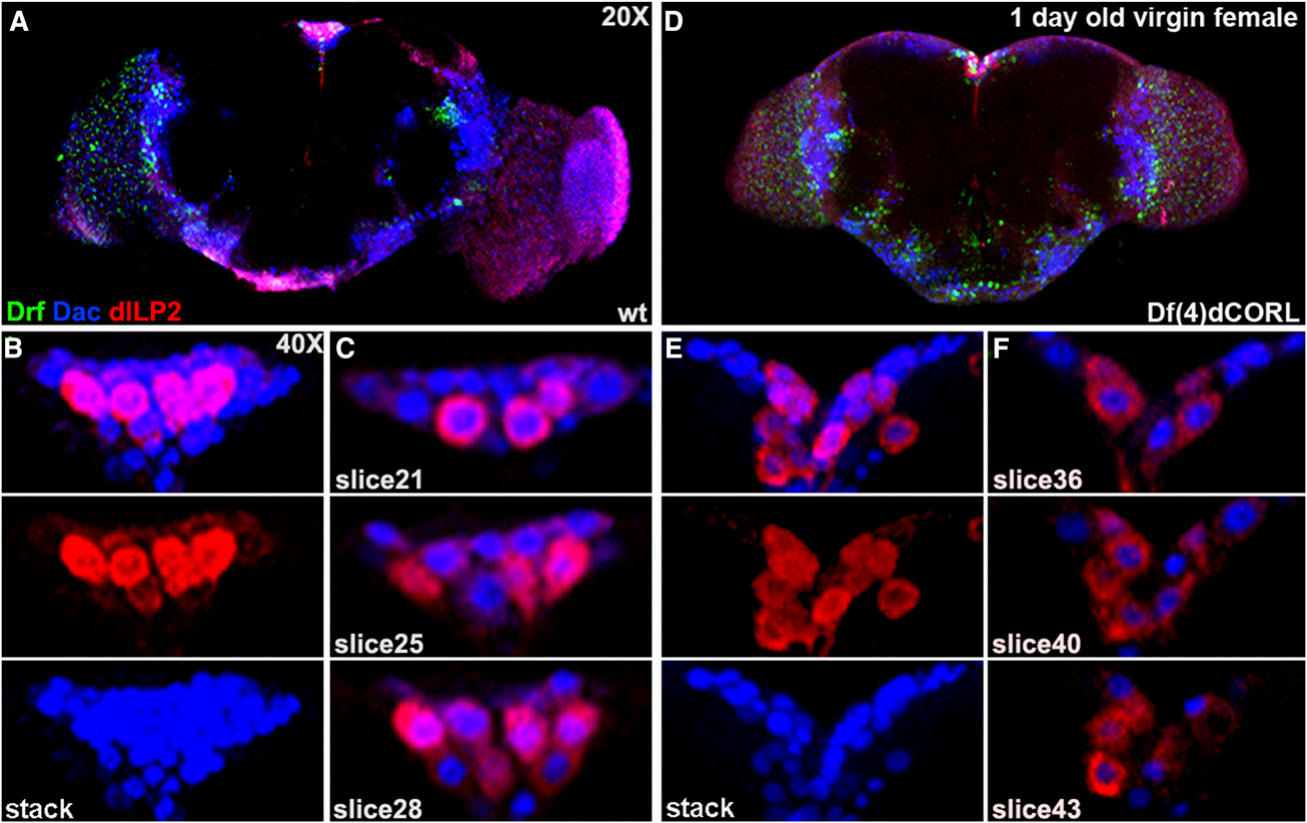
*dCORL* mutant virgin adult brains are missing Dac neurons in the PI. One day old virgin adult female brains in dorsal view with anterior up stained with Drifter (green), Dac (blue - nuclear) and dILP2 (red). A) Wild type (*y^1^w^67c23^*) at 20X shows the presence of all proteins in the PI. B) 40X stack of the PI from the same brain: (top) 2-color view, (middle and bottom) single channel views of dILP2 (red) and Dac (blue). The 2-color view shows dILP2-Dac coexpressing neurons are surrounded by many Dac neurons that form an inverted triangle. C) Three slices covering 8 microns show that all dILP2 neurons express Dac. D) *Df(4)dCORL* brain at 20X shown to scale. All proteins are present in the PI. E) 40X stack of the PI from the same brain: (top) 2-color view, (middle and bottom) single channel views of dILP2 (red) and Dac (blue). The 2-color view shows that there has been a modest decrease in the number of dILP2-Dac coexpressing neurons and a substantial decrease in Dac neurons. The dILP2-Dac coexpressing neurons and the majority of remaining Dac neurons form a single row along the apical surface of the PI (with the exception of a single dILP2 cell). The absence of additional dILP2-Dac and Dac only neurons medially has caused the apical row to collapse into a V-shape. F) Three slices covering a span of 8 microns shows that all dILP2 neurons express Dac.

We then assayed Dac expression in one day old virgin adult female brains as a surrogate for dILP5, another family member produced in neurosecretory cells of the PI. In wild type, Dac expressing neurons are much more numerous than either dILP2 or Drifter, but the topology of Dac cells matches dILP2 by forming an inverted triangle pointing medially (Figure 5A,B). In addition, every dILP2 neuron also expresses Dac (Figure 5C). In *dCORL* mutant brains, Dac neurons display a similar phenotype to dILP2. There is a substantial reduction in the number of Dac expressing cells and the inverted pyramid of Dac cells has largely collapsed into a single row forming a V-shape (Figure 5D,E). In these brains every dILP2 neuron also expresses Dac (Figure 5F). The data suggests that in *dCORL* mutants there is also an effect on dILP5 neurons.

**Figure 6:**
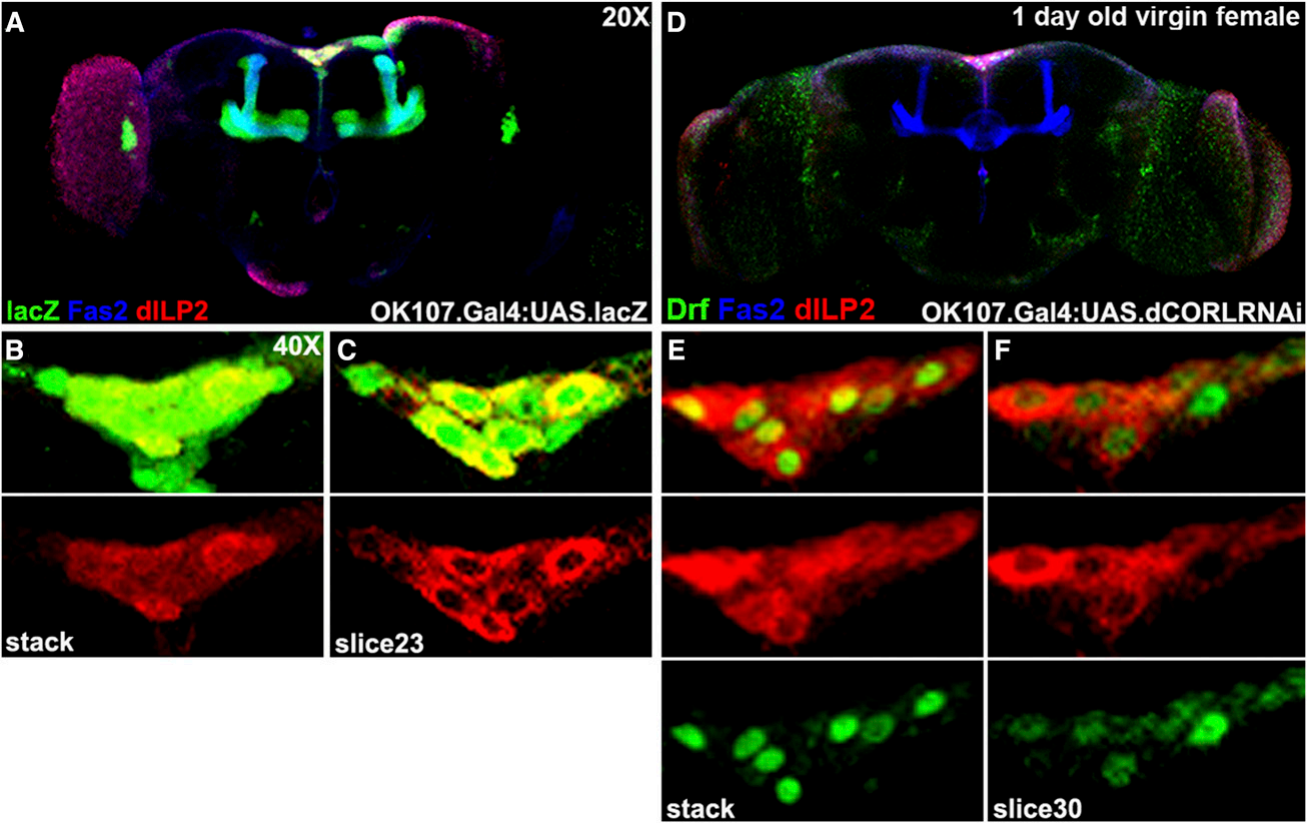
dCORL-RNAi in the PI phenocopies the *Df(4)dCORL* dILP2 phenotype. One day old virgin adult female brains in dorsal view with anterior up. A) Wild type (*y^1^w^67c23^*) with OK107.Gal4 driving UAS.lacZ at 20X displaying lacZ (green), Fas2 (blue) and dILP2 (red) shows the presence of lacZ with Fas2 in the mushroom body lobes and lacZ with dILP2 in the PI. B) 40X stack of the PI from the same brain: (top) 2-color view, (middle) single channel view of dILP2 (red). Both views show the wild type pyramidal structure of lacZ and dILP2 expressing neurons in the PI. C) One slice showing the same topology. D) Wild type (*y^1^w^67c23^*) with OK107.Gal4 driving UAS.dCORL-RNAi displaying Drifter (green), Fas2 (blue) and dILP2 (red). Mushroom body defects due to loss of dCORL in *Df(4)dCORL* adult brains are visible that verify dCORL-RNAi expression. E) 40X stack of the PI from the same brain: (top) 2-color view, (middle and bottom) single channel views of dILP2 (red) and Drifter (green). These views show that there has been a modest decrease in the number of dILP2 expressing neurons and that their topology has flattened out though not to the extent seen in *Df(4)dCORL*. F) One slice shows that all dILP2 neurons express Drifter, a phenocopy of the *Df(4)dCORL* dILP2 phenotype.

To assess whether it was the loss of dCORL within Df(4)dCORL that led to the dILP2 and Dac/dILP5 phenotypes we examined OK107.Gal4 driving UAS.dCORL-RNAi in otherwise wild type one day old virgin adult female brains. Our control for OK107.Gal4 expression was UAS.lacZ and our control for UAS.dCORL-RNAi was adult mushroom body lobe defects previously shown to be due to the loss of dCORL (Takaesu *et al.* 2012). As expected, expression of lacZ had no effect on the mushroom body (Figure 6A). There was also no effect on the topology or number of dILP2 cells in the PI (mean 16.3, n = 4; Figure 6B,C). In the dCORL-RNAi brains mushroom body defects were plainly visible (Figure 6D). In the PI, phenotypes resembling *Df(4)dCORL* were observed. First, the pyramidal shape of dILP2 neurons was not completely lost but the majority of dILP2 neurons formed a row along the apical surface. Second, there was a reduced number of dILP2 neurons (mean 13.0, n = 4; Figure 6E,F). Third and most importantly, all of the dILP2 neurons expressed Drifter as those lacking Drifter were absent. It is the commonality of this latter phenotype between *Df(4)dCORL* and dCORL-RNAi that establishes this phenotype as *dCORL* dependent.

Taken together, we conclude from these loss of function experiments that *dCORL* plays a role in determining the number of dILP2 and Dac/dILP5 cells in the larval and adult PI. Further, *dCORL* appears to influence the identity of larval and adult dILP2 cells, as when it is lost so are dILP2 cells that do not express Drifter.

### dCORL mutant virgin adult longevity defects are fully rescued by mating in both sexes

To determine whether any adult lifecycle traits were affected by the loss of this subset of dILP2 expressing neurons we examined fecundity, fertility and longevity (as virgins and mated) in *dCORL* mutant males and females. In addition to the *yw* parental strain, six control lines were employed to eliminate the possibility that observed phenotypes for *Df(4)dCORL* mutant adults were due to loss of one of the other three genes in the deletion. These were *Pbac{WH}^f07015^*, *Glu-RA^112b^*, *Glu-RA^2b^*, *Pbac{WH}^f06253^*, *Pbac{RB}^e02096^* and *spx^720RW^* (Takaesu *et al.* 2012). Tested alleles for two of the three genes are demonstrated nulls: *Glu-RA^112^* (Bogdanik *et al.* 2004) and *spx^720RW^* (Dai *et al.* 2008). For the third gene, *Pbac{RB}^e02096^* is expected to disrupt all predicted transcripts of CG32016 and thus should also serve as a null. We showed previously the *toy* transcription unit is not affected in the *dCORL* deletion (Takaesu *et al.* 2012). We recently found that Toy expression is unaffected in *Df(4)dCORL* larvae and adults (Tran *et al.* 2018). Assays of fecundity and fertility will be reported elsewhere.

Assays of longevity turned out quite surprisingly. Studies of virgins showed a statistically significant reduction in lifespan for *dCORL* mutants *vs.* the parental *yw* strain. For reference, note that the *dCORL* mutant strain has a *yw* background and thus we use *yw* as a control in these experiments just as in our expression studies. While the *yw* strain has a lifespan roughly 50% shorter than the wild type strains OregonR and Canton-S (Grandison *et al.* 2009), it is the relative lifespans of matched *Df(4)dCORL* flies to *yw* flies that is important. Overall, a majority of the six control lines have *yw* backgrounds. Genotypes and an initial set of longevity data for eight lines (*Df(4)dCORL*, *yw* and the six control lines) are described in Table S1.

**Figure 7:**
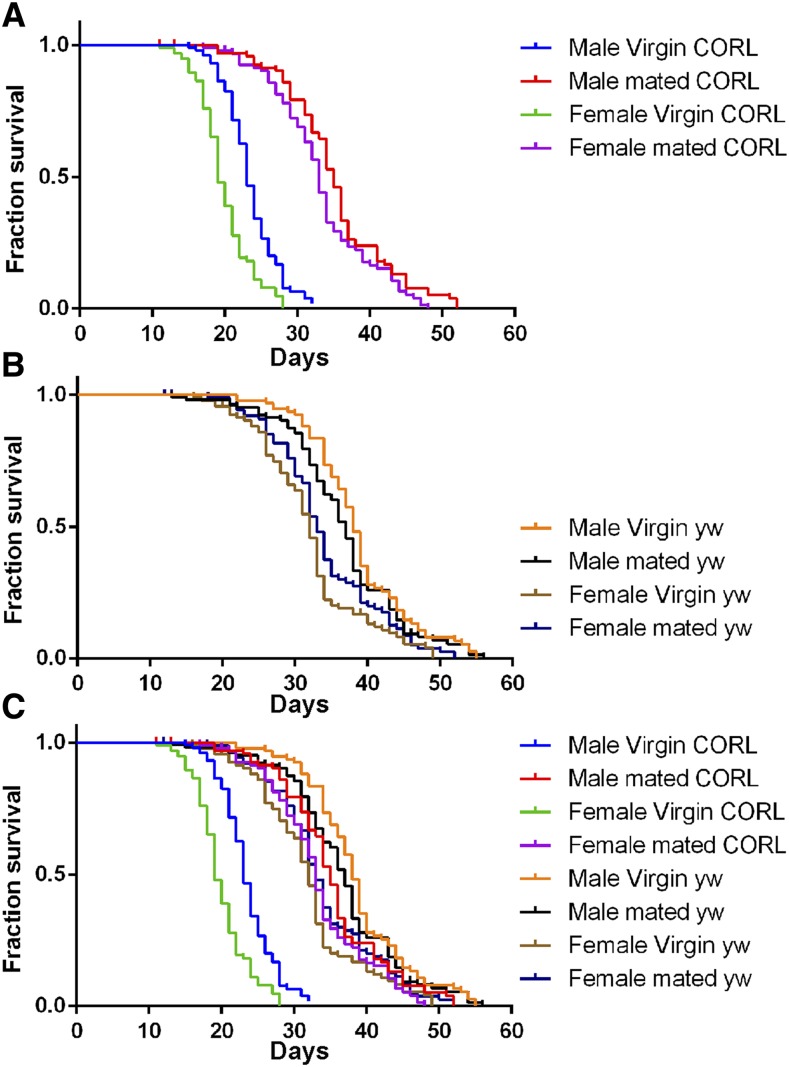
*dCORL* mutant virgin adult longevity defects are fully rescued by mating in both sexes. Cohorts of 100 flies of each genotype and mating status were analyzed in parallel under identical conditions. Mortality assessments were made daily. Numerical data from this experiment is in Tables 1 and 2 with a much larger set of genotypic controls shown in Table S1. Note that *Df(4)dCORL* has a *yw* background. A) *Df(4)dCORL* virgins display a relatively short lifespan but upon mating there is a highly significant extension of lifespan for both sexes. B) *yw* virgins and mated flies of both sexes show no lifespan differences. C) *Df(4)dCORL* virgins of both sexes show a highly significant lifespan reduction *vs. yw*. Upon mating, the lifespan reduction of *Df(4)dCORL* virgins is completely rescued as mated *Df(4)dCORL* adults of both sexes are not different from *yw*.

We repeated the longevity studies with much larger cohorts of *dCORL* mutant and *yw* adults. For *dCORL* mutants, virgin females lived roughly 19.5 days (Figure 7A). For *yw*, virgin females lived on average 31.7 days (Figure 7B). *dCORL* mutant virgin females live only 62% as long as *yw* virgin females (Figure 7C), a highly significant reduction (*yw vs. dCORL* mutant *P* < 1.0x10^−6^, Table 1). For *yw*, sibmated females lived roughly 33.9 days (an insignificant increase of 07% over *yw* virgin females; *P* = 0.14, Table 1). For *dCORL* mutants, sibmated females lived on average 33.0 days (an increase of 64% over *dCORL* mutant virgin females). The increase in lifespan for virgin *vs.* mated *dCORL* mutant females is highly significant (*P* < 1.0x10^−6^, Table 1) and nearly 10-fold greater than the longevity increase of *yw* virgin *vs. yw* mated females. The lifespan of *dCORL* mutant mated females is not significantly different from *yw* mated females (*P* = 0.57; Table 1). The highly significant reduction in *dCORL* mutant virgin female lifespan in comparison to *yw* virgin females is fully rescued by mating; *dCORL* mutant mated females have the same lifespan as *yw* mated females (Figure 7C).

The mating induced lifespan increase for *dCORL* mutant males is comparable. For *dCORL* mutants, virgin males lived roughly 23.2 days (Figure 7A). For *yw*, virgin males lived on average 38.3 days (Figure 7B). *dCORL* mutant virgin males live 61% as long as *yw* virgin males (Figure 7C), a highly significant reduction (*yw vs. dCORL* mutant *P* < 1.0x10^−6^, Table 1). For *yw*, sibmated males lived roughly 36.4 days (an insignificant decrease of 05% from *yw* virgin males, *P* = 0.08, Table 1). For *dCORL* mutants, sibmated males lived on average 35.0 days (an increase of 51% over *dCORL* mutant virgin males). The increase in lifespan for virgin *vs.* mated *dCORL* mutant males is highly significant (*P* < 1.0x10^−6^, Table 1) and starkly different from the insignificant longevity decrease of *yw* virgin *vs. yw* mated males. The lifespan of *dCORL* mutant mated males is not significantly different from *yw* mated males (*P* = 0.07, Table 1). The highly significant reduction in *dCORL* mutant virgin male lifespan in comparison to *yw* males is fully rescued by mating; *dCORL* mutant mated males have the same lifespan as *yw* mated males (Figure 7C).

Application of the log-rank test (Mantel-Cox) optimized for survival studies arrives at the same statistical significance. Lifespan of *dCORL* mutant virgins are highly significantly shorter than *yw* virgins (males *P* < 1.0x10^−6^, females *P* < 1.0x10^−6^, Table 2). Lifespan of *dCORL* mutant mated adults are highly significantly longer then their virgin counterparts (males *P* < 1.0x10^−6^, females *P* < 1.0x10^−6^, Table 2). Lifespan of *yw* mated adults is not significantly different from their virgin counterparts (males *P* = 0.21, females *P* = 0.08, Table 2). Lifespan of *dCORL* mutant mated adults are not significantly different from their *yw* counterparts (males *P* = 0.07, females *P* = 0.39, Table 2). Application of statistical tests to median lifespans, rather than means as noted above, does not impact the p values in any meaningful way (Table S2). The fact that mating can induce the complete reversal of the lifespan defect in *dCORL* mutant virgins of both sexes is unprecedented.

### dILP2 neurons lacking Drifter are fully rescued by mating in dCORL mutant adult brains

**Figure 8:**
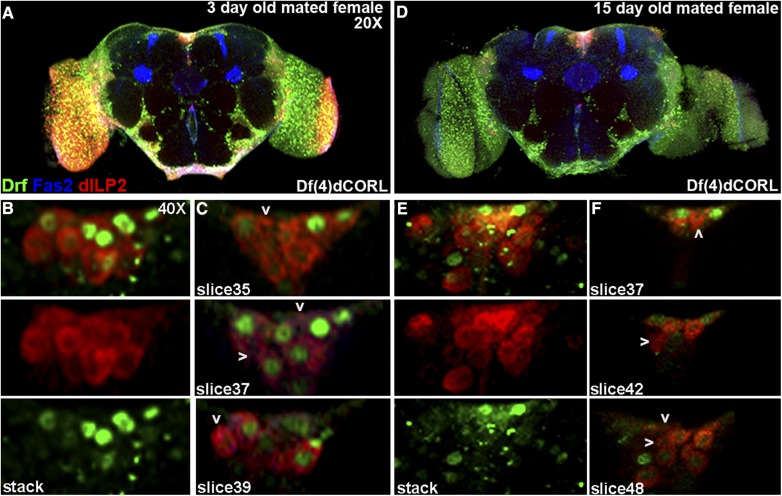
dILP2 neurons lacking Drifter are fully rescued by mating in *dCORL* mutant adult brains. *Df(4)dCORL* mated adult female brains shown as in Fig. 4. A) Three day old at 20X shows rescue of PI topology compared to *dCORL* mutant virgin females. B) 40X stack of the PI from the same brain: (top) 2-color view, (middle and bottom) single channel views of dILP2 (red) and Drifter (green). Three day old mated females display an increase in dILP2 expressing neurons *vs.* virgin females due to the presence of rescued neurons expressing dILP2 only. C) Three slices at 40X covering 10 microns show the pyramidal structure of the PI is similar to wild type though more compact. Multiple dILP2 neurons lacking Drifter are present (white arrowheads). D) Fifteen day old at 20X shows that the rescue of PI topology persists. E) 40X stack of the PI from the same brain: (top) 2-color view, (middle and bottom) single channel views of dILP2 (red) and Drifter (green). Fifteen day old mated female also displays an increase in dILP2 expressing neurons *vs.* virgins indicating that rescued neurons expressing dILP2 only persist. F) Three slices covering a span of 24 microns show the pyramidal structure of the PI is similar to wild type though more expansive. Multiple rescued dILP2 neurons lacking Drifter (white arrowheads) are present after two weeks.

In an attempt to understand the basis for the longevity rescue we examined dILP2 and Drifter expression in the brains of sibmated three day old and fifteen day old *dCORL* mutant adult females (brain size data in Table S3). Right away we noted that the topology of the PI was restored that and that this was due to the presence of dILP2 neurons lacking Drifter (Figure 8A,B). Closer examination showed that the arrangement of Drifter expressing neurons in the PI was not completely wild type. There were 2-3 dILP2-Drifter coexpressing neurons in the medial triangle that normally contained dILP2 only neurons. Nevertheless, the number of Drifter neurons in the three day old mated mutants was not significantly different from virgin wild type or virgin *dCORL* mutants (10.3 mean, n = 7; *vs.* virgin *dCORL* mutants *P* = 0.45, Table 3). Analysis of five individual slices covering a span of 10 microns confirmed the rescue of the inverted pyramidal 3-dimensional structure of the PI (albeit more compact) and that this was due to the presence of dILP2 only neurons medially (Figure 8C). The average number of dILP2 neurons was increased in mated *vs.* virgin mutants but not with statistical significance again due to the large standard error (mean 13.6 for three day mated *vs.* 11.4 for virgin; *P* = 0.39, Table 3).

**Table 3:** Missing dILP2 cells in *dCORL* mutant virgin adults are fully rescued by mating

Genotype, virgin/mated days post-eclosion	n	dILP2 PI cell counts	*P* value *vs. yw* virgin	*P* value *vs. Df(4)* virgin	*P* value *vs. Df(4)* 3 day mated
*yw*, virgin	6	16.7 ± 1.8			
*Df(4)*, virgin, 1 day	8	11.8 ± 4.3	0.075		
*Df(4)*, mated, 3 day	7	13.6 ± 4.0	0.248	0.445	
*Df(4)*, mated, 15 day	8	12.8 ± 2.8	0.083	0.611	0.673
		Drifter PI cell counts			
*yw*, virgin	6	9.8 ± 1.1			
*Df(4)*, virgin, 1 day	8	8.9 ± 5.1	0.702		
*Df(4)*, mated, 3 day	7	10.3 ± 6.3	0.883	0.663	
*Df(4)*, mated, 15 day	8	8.4 ± 3.5	0.439	0.832	0.262

Observations in mated fifteen day old adult *dCORL* mutants showed that the atypical presence of Drifter neurons medially persists (Figure 8D,E). The number of Drifter neurons remains not significantly different from any other analyzed groups (mean 8.4, n = 8; *vs.* mated three day old mutants *P* = 0.51, Table 3). Analysis of twelve individual slices covering a span of 24 microns revealed a spatial expansion of the inverted pyramidal 3-dimensional structure of the PI in comparison to three day old mated mutants (Figure 8F). The number of dILP2 only neurons in the PI of mated fifteen day old adult *dCORL* mutants was essentially unchanged from three day old mated mutants (mean 12.8, n = 8; *P* = 0.67, Table 3). Overall the data from mated *dCORL* mutants reveals that after the rapid rescue of dILP2 neurons in the PI by mating (within three days), these neurons are maintained over at least the next twelve days.

## Discussion

The data supporting a connection between *dCORL* expression and function, insulin neurons, longevity and mating are: 1) *dCORL* reporter expression in all dILP2 neurons of the larval and adult PI, 2) *dCORL* mutant virgin adults display significant lifespan reduction and have lost all dILP2 neurons lacking Drifter in their PI, and 3) *dCORL* mutant adult lifespan is fully rescued by mating as are dILP2 neurons lacking Drifter in their brains.

We attribute the phenotypes of *Df(4)dCORL* to loss of *dCORL* for three reasons. First, the presence of the *dCORL* reporter AH.lacZ in all dILP2 cells of the larval and adult brain that are the same cells affected in *Df(4)dCORL* brains. While circumstantial, Tran *et al.* (2018) further illustrates the fidelity of AH.lacZ and *dCORL* brain expression (*e.g.*, AH.lacZ does not overlap with neighboring Toy in larvae or adults). Second, the published brain expression patterns for *Glu-RA* (anterior *ad* neurons in larvae and antennal+optic lobes in adults; Ramaekers *et al.* 2001) and for *sphinx* (Chen *et al.* 2011; antennal lobes in larvae and the *ab1* class of large basiconic sensillum of the antenna in adults) do not correspond to insulin producing cells. No images of CG32016 brain expression are available, but two lines of evidence suggest it is not present in larval or adult insulin producing cells. It was found in a cell-based overexpression screen to bind Orb2 (a mushroom body specific protein; White-Grindley *et al.* 2014) and in an adult odor aversion RNAi screen (Walkinshaw *et al.* 2015). Third, and most importantly the phenocopy of the *Df(4)dCORL* dILP2 phenotype by expression of dCORL-RNAi in the PI.

It was shown some time ago that mating reduces female lifespan in Drosophila due to exposure to sex-peptide in the male ejaculate (Chapman *et al.* 1995). Yet *Df(4)dCORL* exhibits the opposite phenotype. In the 20+ years since the original sex-peptide paper, numerous reports have shown the situation is more complex than initially described. For example, Fricke *et al.* (2010) showed that diet altered the presence, magnitude and sign of the effects of sex-peptide on a variety of phenotypic traits including lifespan. Altering the “sign” of the effect means that lifespan was increased by sex-peptide under some circumstances. In an second example, Grandison *et al.* (2009) reported that the Dahomey strain utilized in the 1995 paper is among the longest-lived wild type lines in the lab; Dahomey lifespan is more than 40% longer than OregonR and Canton-S. Effects of mating on this outlier strain may not be readily transferrable to strains such as *Df(4)dCORL*. Further, the fact that *Df(4)dCORL* males and females are similarly affected suggests a sex-peptide independent mechanism.

The fact that there are likely several mechanisms connecting mating and longevity is a conclusion of Fricke *et al.* (2010) and connections between mating and longevity independent of insulin have been seen (*e.g.*, Bowman and Tatar 2016). Alternatively, there are mutants affecting the insulin signaling pathway that extend lifespan independent of mating (*e.g.*, Huang *et al.* 2015), but no previous data has connected insulin, longevity and mating. If *dCORL* regulates insulin producing neuron formation, maintenance, identity or function downstream of TGF-β/Activin signaling (it modulates Activin signaling in the mushroom body), that suggests a mechanism for the connection. For example, Activin is a major metabolic regulator via functional links with insulin and dFOXO and these interactions influence longevity (Bai *et al.* 2013; Ghosh and O’Connor 2014). Also midgut-derived Activin elicits responses in the fat body that modulate the metabolism of sugars and triglycerides (Song *et al.* 2017a,b). The hypotheses that *dCORL* function in the PI is influenced by, or contributes to Activin signaling, are currently being tested. A TGF-β independent function for *dCORL* in the insulin pathway is also possible, as suggested by the TGF-β independent role of *dSno* in Wg signaling (Quijano *et al.* 2010).

Another intriguing issue is the mechanism that allows *dCORL* mutant virgin longevity defects to be fully rescued by mating in both sexes. Two papers have shown a connection between changes in female reproductive status (*i.e.*, pre- and post-mating) and intestinal remodeling, presumably to more efficiently nourish the growth of oocytes (Cognigni *et al.* 2011; Reiff *et al.* 2015). The second paper identifies Juvenile Hormone as an integral part of the process. The presence of Juvenile Hormone in both sexes provides a point of departure for characterizing the mechanism of interaction between dCORL, insulin, longevity and mating. One hypothesis for *dCORL* mutant longevity reversal is that a gender-neutral mating-responsive neural network employs Juvenile Hormone in response to reproductive activity to influence insulin neuron identity in the PI.

The rapid rescue of dILP2 neurons lacking Drifter in *dCORL* mutant adults (within three days of mating) lends itself to two hypotheses. The first hypothesis is that dILP2 only neurons are never actually lost in *dCORL* mutants. What is lost is dILP2 expression in non-Drifter expressing PI neurons. In this case what is rescued by mating is dILP2 expression. Alternatively, rescued dILP2 only neurons are born via adult brain neurogenesis triggered by mating in *dCORL* mutants. The neurogenesis hypothesis is consistent with the observed regeneration of PI topology in mated *dCORL* mutants. The possibility of adult neural regeneration in the brain of *dCORL* mutants leads to the essentially unexplored area of adult brain neuroblast division in Drosophila. A Flybase search identified only a single paper describing the activation of adult brain neuroblast cell division in response to acute damage (Fernández-Hernández *et al.* 2013). In contrast there is widespread interest in understanding the origins of adult born neurons in the mammalian brain (*e.g.*, Ernst *et al.* 2014; generation of striatal neurons in adult humans). Interest in mammalian adult neurogenesis is stimulated by the potential for harnessing this process therapeutically in traumatic brain injury and disease (*e.g.*, Iqbal *et al.* 2014). For example, dopamine derived from adult born striatal neurons regulates systemic glucose metabolism in humans and stimulation of this process reduced the insulin dependence of a diabetic patient (ter Horst *et al.* 2018). *dCORL* mutants may provide a non-invasive, genetic model for analyses of adult brain neurogenesis and any connections to the insulin signaling pathway. A link between insulin signaling and ventral cord neuroblast reactivation after hatching has been reported (Otsuki and Brand 2018).

Our *dCORL* mutant brain and longevity data generate numerous hypotheses for our colleagues studying human and mouse SKOR1/2 regarding their function in neural development and adults. During development, the coincidence of SKOR1/2 and CNS specific homologs of Drifter could be examined (POU3F2 and POU3F4). In adults, while the brain is not the site of insulin production, certainly there are signals from the brain that influence insulin signaling and/or longevity (*e.g.*, Templeman *et al.* 2017). An relationship between insulin and longevity in humans is shown by the reduced lifespan of patients with insulin resistance syndrome (*e.g.*, Ranieri *et al.* 2013). One approach could be genome wide association studies for connections between insulin resistance or longevity and single nucleotide polymorphisms in hSKOR1/2.

In summary, our data on *dCORL* expression and function suggest this gene participates in a previously unknown neural network connecting the insulin signaling pathway, longevity and mating. The conserved sequence and CNS specificity of all CORL proteins plus the obvious adaptive benefit to the offspring of parents living longer implies that this network exists in mammals. Further studies of CORL family members will advance our understanding of their expression and function relevant to human homeostasis and disease.
